# Supplementary material for: Mode conversion of hyperbolic phonon polaritons in van der Waals terraces
Source: Nat Commun. 2025 Dec 30;17:1273. doi: 10.1038/s41467-025-68030-7 (PMC12868863; doi:10.1038/s41467-025-68030-7)
Supplement: Supplementary file 1 — Supplementary Information [file 41467_2025_68030_MOESM1_ESM.pdf]

Supplementary Information: Mode Conversion of Hyperbolic Phonon Polaritons in van der Waals terraces

Byung-Il Noh[1†], Sina Jafari Ghalekohneh[2†], Mingyuan Chen[1], Jialiang Shen[1], Eli Janzen[3], Lang Zhou[1], Pengyu Chen[1], James Edgar[3], Bo Zhao[2]*, and Siyuan Dai[1]*

[1]*Materials Research and Education Center, Department of Mechanical Engineering, Auburn University, Auburn, Alabama 36849, USA*

[2]*Department of Mechanical Engineering, University of Houston, Houston, Texas, 77204, USA*

[3]*Tim Taylor Department of Chemical Engineering, Kansas State University, Manhattan, Kansas 66506, USA*

*Correspondence to: sdai@auburn.edu and bzhao8@uh.edu

[†]These authors contribute equally

Supplementary Note 1. Standing wave interference period ρ for the $l = 0 \rightarrow 1$ converted polaritons

The fringe period ρ of the $l = 0 \rightarrow 1$ converted polaritons is determined by the phase difference between two interfering waves that contribute to the standing wave oscillations. In the Fourier Transform (FT) spectra in Figures 2c-d in the main text, the $\Delta = 35\ \mu m^{-1}$ resonances correspond to standing wave interference between the edge-converted first-order (index $l = 1$) and the newly launched zeroth-order ($l = 0$) polaritons, see the schematic in Supplementary Figure 1. Assuming the tip-launched polaritons have an initial electric field E_0 (right under the tip). The accumulated phases in Supplementary Figure 1 are $k_0'(L_{\text{tip}} + L) + k_1'L + \phi$ for the $l = 0 \rightarrow 1$ converted wave and $k_0'L_{\text{tip}}$ for the newly-launched $l = 0$ wave, respectively. Following the definition in the main text, here $k_1' = 2\pi/\lambda_l$ is the real momentum of the l-th order polaritons. L_{tip} and L are the distances to the tip and edge, respectively. ϕ is the phase change during the mode conversion (reflection) at the step-shape edge. At interference maxima (fringes):

$$k_0'(L_{tip} + L) + k_1'L + \phi - k_0'L_{tip} = (k_0' + k_1')L + \phi = 2\pi N. \quad \text{(S1)}$$

$N = 0, 1, 2, \ldots$ are integers. Therefore, the fringe period is $\rho = 2\pi / (k_0' + k_1')$. In the FT spectrum, the resonance is $\Delta = k_0' + k_1'$.

Supplementary Note 2. Decomposition of the s-SNOM data via FT analysis

This section provides the decomposition of a representative s-SNOM line profile (Supplementary Figure 2a, reproduced from Figure 2c in the main text) into its constituent real-space oscillation components based on the corresponding FT resonances (Supplementary Figure 2a). In Supplementary Figure 2b, we perform inverse FT transforms using the filtered resonances marked in red, blue, and pink in Supplementary Figure 2a. They correspond, respectively, to standing wave interferences for edge-launch-photon-interfere + tip-launch-edge-reflect $l = 0$ polaritons (red), $l = 0 \rightarrow 1$ converted polaritons (blue), and tip-launch-edge-reflect $l = 1$ polaritons (pink). In comparison to the s-SNOM line profile (black curve in Supplementary Figure 2b), it is evident that the s-SNOM long-period fringes arise from the $l = 0$ polaritons, while the short-period beats originate from the $l = 0 \rightarrow 1$ converted polaritons (blue). In contrast, the tip-launch-edge-reflect $l = 1$ polaritons correspond to the pink resonance that is orders of magnitude weaker than the red and blue ones (Supplementary Figure 2a) and their real-space features (pink curve, Supplementary Figure 2b) cannot be observed from the s-SNOM line profile (black). This conclusion is further confirmed in Supplementary Figure 2c: the orange curve, obtained by summing only the $l = 0$ and $l = 0 \rightarrow 1$ components, already reproduces all observable features of the experimental data. Adding the tip-launch-edge-reflect $l = 1$ component (green curve) does not introduce any additional visible features, confirming its negligible contribution.

Supplementary Note 3. Finite-difference frequency-domain (FDFD) simulations

The finite-difference frequency-domain (FDFD) method is utilized to analyze the mode conversion by assessing the reflection coefficient of electromagnetic waves reflected from the hexagonal boron nitride (hBN) simple slab and the step-shape terrace. We initially use the FDFD method to calculate the various eigenmodes of the simple slab hBN, which are subsequently employed as the source in our simulation. To calculate the eigenmodes in a simple slab hBN, we need to find the approximated eigenvalues, which are the corresponding tangential wavevector k_x,

to the FDFD eigenmode solver. These eigenvalues can be obtained by solving the hyperbolic polariton dispersion in a simple slab hBN[1]:

$$k_x(\omega) = -\frac{\Psi}{d}\left[\arctan\left(\frac{1}{\varepsilon_t\Psi}\right) + \arctan\left(\frac{\varepsilon_{SiO_2}}{\varepsilon_t\Psi}\right) + \pi l\right],\ \Psi = \frac{\sqrt{\varepsilon_z}}{i\sqrt{\varepsilon_t}}, \quad \text{(S2)}$$

where ω is the frequency, d is the thickness of the hBN slab, and l is the mode index (l = 0, 1, 2, …). $\varepsilon_t=\varepsilon_x=\varepsilon_y$ and ε_z are in-plane and out-of-plane permittivities of hBN. Using the Lorentz model, the permittivity of hBN can be written as:

$$\varepsilon_x = \varepsilon_{\infty,x}\left(1 + \frac{\omega_{\mathrm{LO},x}^2 - \omega_{\mathrm{TO},x}^2}{\omega_{\mathrm{TO},x}^2 - i\gamma_x\omega - \omega^2}\right) \quad \text{(S3)}$$

$$\varepsilon_z = \varepsilon_{\infty,z}\left(1 + \frac{\omega_{\mathrm{LO},z}^2 - \omega_{\mathrm{TO},z}^2}{\omega_{\mathrm{TO},z}^2 - i\gamma_z\omega - \omega^2}\right) \quad \text{(S4)}$$

Here $\varepsilon_{\infty,x}$ = 4.87 and $\varepsilon_{\infty,z}$ = 2.95. $\omega_{\mathrm{LO},x}$ = 1610 cm^{-1} and $\omega_{\mathrm{LO},z}$ = 830 cm^{-1} are the in-plane and out-of-plane longitudinal optical (LO) phonon frequency, and $\omega_{\mathrm{TO},x}$ = 1370 cm^{-1} and $\omega_{\mathrm{TO},z}$ = 780 cm^{-1} are the in-plane and out-of-plane transverse optical (TO) phonon frequency[2]. γ_x = 5 cm^{-1} and γ_z = 4 cm^{-1} are the in-plane and out-of-plane damping coefficients.

Using Eq. S2, we can simulate the polariton field distribution at each hyperbolic branch via the FDFD eigenmode solver. Figures 4a–d in the main text display the x-component of the electric field (E_x) for the first four modes (l = 0, 1, 2, and 3) in a 34 nm-thick hBN slab. In Supplementary Figures 3a-d, we plot the normalized E_x distribution in false-color maps when those modes are launched and then propagate in the hBN slab.

In Supplementary Figure 4, FT spectra of the reflected fields for the simple slab (symmetric, Supplementary Figure 4a) and step-shape (asymmetric, Supplementary Figure 4b) edges reveal hyperbolic polariton mode conversions. First, we put the source (black solid line) before the edge of the hBN slab and then let the initialized field propagate towards the edge (to the right). Then, we capture the reflected field behind the source (on the left of the source), which presents the reflected field only. In Supplementary Figures 4c-e and Supplementary Figures 3f-h, we plot the FT spectra of the reflected field at various incident modes (l = 1, 2, and 3) for the simple slab and step-shape edge, respectively. Similar to the l = 0 incidence in the main text (Figures 4f and 4h), the simple slab edge preserves the modal symmetry: polaritons are mainly reflected at the same l (Supplementary Figures 4c-e). In contrast, the step-shape edge does not preserve the modal symmetry but causes evident mode conversions: polaritons are reflected at different l (Supplementary Figures 4f-h).

To quantify the reflected polaritons at each l, we utilize mode orthogonality to calculate their reflection coefficient r. The mode orthogonality arises from power conservation when a wave propagates through a waveguide. To preserve power conservation, the integral of the real component of the complex Poynting vector across the cross-section of the waveguide must remain constant. Therefore, as the wave in our system propagates in the x-direction, we have:

$$\frac{d}{dx}\int dydz\mathrm{Re}\ (E \times H^*) \cdot \hat{x} = 0. \quad \text{(S5)}$$

E and H are the total electric and magnetic fields. H^* is the complex conjugate of the magnetic fields. The electric and magnetic fields of the multi-mode waveguide can be expressed as a linear superposition of modes:

$$E(x,y,z) = \sum_l a_l e^{-jk_{x,l}y} \cdot e_l(x,z) \quad \text{(S6)}$$

$$H(x,y,z) = \sum_l a_l e^{-jk_{x,l}y} \cdot h_l(x,z). \quad \text{(S7)}$$

$e_l(x,z)$, $h_l(x,z)$, and $k_{x,l}$ are the electric, magnetic field, and tangential momentum of the l-th order mode (l = 0, 1, 2, 3, …), respectively. a_l is the amplitude of each eigenmode. By combining Eqs. S5, S6, and S7, we obtain the orthogonality relation between modes of different orders ($m \neq n$) as:

$$\int dydz(e_m \times h_n^* + e_n^* \times h_m) \cdot \hat{x} = 0 \quad \text{(S8)}$$

Eq. S8 can be combined with normalization requirements to yield:

$$\frac{1}{4}\int dydz(e_m \times h_n^* + e_n^* \times h_m) \cdot \hat{x} = \delta_{mn} \quad \text{(S9)}$$

In our system, we assume that the hBN slab is extended to infinity in the *y*-direction. Therefore, by solving Eq. S9 for transverse magnetic (TM) waves, the orthogonality relation can be expressed as:

$$\int \frac{1}{\varepsilon_z} h_{y,m} h_{y,n}^* \, dz = \frac{2\omega}{k_x} \delta_{mn} \quad \text{(S10)}$$

Where $h_{y,m}$ is the magnetic field of the *m*-th order mode in the *y*-direction. Now, getting back to Eq. S7 and expanding it for the reflected wave by considering that we are studying TM waves, we have:

$$H_{y,ref} = r_0 H_{y,0} + r_1 H_{y,1} + r_2 H_{y,2} + r_3 H_{y,3} + \cdots \quad \text{(S11)}$$

where r_i represents the reflection coefficient for *i*-th order mode. $H_{y,i}$ is the magnetic field of *i*-th order mode in the *y*-direction (i = 0, 1, 2, 3, …). If both sides of Eq. S11 are multiplied by $\frac{1}{\varepsilon_z} H_{y,0}^*$ and integrated over the cross-section of the waveguide, then we have:

$$\int \frac{1}{\varepsilon_z} H_{y,ref}.H_{y,0}^* \, dz = r_0 \int \frac{1}{\varepsilon_z} H_{y,0}.H_{y,0}^* + r_1 \int \frac{1}{\varepsilon_z} H_{y,1}.H_{y,0}^* + r_2 \int \frac{1}{\varepsilon_z} H_{y,2}.H_{y,0}^* + r_3 \int \frac{1}{\varepsilon_z} H_{y,3}.H_{y,0}^* + \cdots \quad \text{(S12)}$$

The right-hand side of Eq. S12 contains only one non-zero term, which is given by $r_0 \int \frac{1}{\varepsilon_z} H_{y,0}.H_{y,0}^*$. The remaining terms on the right-hand side are zero, owing to orthogonality. Consequently, the reflection coefficient of the zero-order mode r_0 is given by:

$$r_0 = \frac{\int \frac{1}{\varepsilon_z} H_{y,ref}.H_{y,0}^* dz}{\int \frac{1}{\varepsilon_z} H_{y,0}.H_{y,0}^* dz} \quad . \quad \text{(S13)}$$

Following a similar method, we can calculate the reflection coefficient for the first-order mode r_1. We multiply both sides of the Eq. S11 by $\frac{1}{\varepsilon_z} H_{y,1}^*$ and integrate it over the cross-section of the waveguide. Therefore, the reflection coefficient for the first-order mode r_1 is:

$$r_1 = \frac{\int \frac{1}{\varepsilon_z} H_{y,ref}.H_{y,1}^* dz}{\int \frac{1}{\varepsilon_z} H_{y,1}.H_{y,1}^* dz} \quad . \quad \text{(S14)}$$

The reflection coefficient of other modes can also be obtained using a similar procedure. Figures 4i-j in the main text present the reflectivity ($R = r^2$) of each mode for the single slab and step-shaped edge structures, respectively. It is worth noting that R_{ij} represents the reflectivity of the *j*-th order mode (j = 0, 1, 2, 3, …) with the incident *i*-th order mode.

To measure the transmission coefficient of different polariton modes for the step-shaped edge, we record the magnetic field of the polaritons ($H_{y,tran}$) at the right side of the hBN terrace (e.g., red dashed line in Supplementary Figure 4b). Using the similar mode orthogonality, the transmission coefficient t_i can be obtained:

$$t_i = \frac{\int \frac{1}{\varepsilon_z} H_{y,tran}.H_{y,i}^* dz}{\int \frac{1}{\varepsilon_z} H_{y,i}.H_{y,i}^* dz} \quad . \quad \text{(S15)}$$

Supplementary Figure 4i presents the transmissivity T (t^2) for different modes. T_{ij} indicates the transmissivity of *j*-th order mode with the incident *i*-th order mode. We note that the integration bounds for the mentioned equations are for the whole *y*-axis, including the air above the hBN and the substrate.

The first column in Supplementary Figure 4i shows that with the incident zeroth-order mode (l = 0), most of the transmitted power remains in the zeroth-order mode. However, when high-order modes (l = 1, 2, 3) are injected, substantial amounts of the transmitted field are in lower-

order modes. For example, injecting the first-order mode results in a considerable amount of transmitted power being in the zeroth-order mode, and injecting the second- or third-order modes result in the first-order mode being dominant in the transmitted fields. This phenomenon can be explained based on the dispersion relation (Eq. S2). When the thickness of the hBN changes, the polariton momentum varies accordingly. Decreasing thickness causes an increase in the mode momentum. Thus, the incident polariton momentum on the thicker side is similar to that of a lower-order mode on the thinner side. Consequently, injecting a first-order mode results in a substantial zeroth-order mode transmitted, and injecting second- or third-order modes results in the first-order mode being predominantly transmitted. When the zero-order mode is injected, the dominant mode in the transmitted field remains in the zeroth-order mode because no lower modes are available.

Supplementary Note 4. FT analysis of the step-size dependent s-SNOM data

Similar to Figures 2 and 3 in the main text, polariton mode conversion can be analyzed by FT of the step-size dependent s-SNOM data in Figure 5. Supplementary Figures 5a-d are the FT spectra of the s-SNOM line profiles in Figure 5d of the main text for hBN terraces with the step ratios $h/H = 0.16$, 0.33, 0.67, and 0.79. All FT spectra exhibit similar resonances at $\Delta < 5\ \mu m^{-1}$ and $\Delta \sim 23\ \mu m^{-1}$. The FT resonances at $\Delta < 5\ \mu m^{-1}$ correspond to fringes of the $l = 0$ polaritons. The $\Delta \sim 23\ \mu m^{-1}$ resonances correspond to fringes of the converted $l = 0 \rightarrow 1$ polaritons. In Figure 5e of the main text, the line profiles of the converted $l = 0 \rightarrow 1$ polaritons were plotted by inverse FT[3, 4] of the resonances at $\Delta \sim 23\ \mu m^{-1}$ for each h/H.

Supplementary Note 5. The extraction of $l = 0 \rightarrow 1$ polariton mode conversion rate R_{01}

The $l = 0 \rightarrow 1$ polariton mode conversion rate R_{01} can be extracted by measuring the intensity A of the polariton fringe oscillations. This oscillation intensity A can be quantified by fitting the $l = 0 \rightarrow 1$ s-SNOM line profiles (Figure 5e) with the envelope of a sinusoidal wave function Ae^{kL}[5, 6]. For example, the $l = 0 \rightarrow 1$ polariton line profile from the hBN terrace with a step ratio $h/H = 0.33$ can be fitted with $A = 0.26$ (Supplementary Figure 6). The mode conversion rate R_{01} can be obtained by normalizing the A at each h/H to that of the reflected $l = 0$ mode at the simple slab edge, which possesses most power of the incident $l = 0$ mode. The absolute R_{01} can be extracted (Figure 5f) by leveraging the simulated $R_{00} = 0.71$.

Supplementary Note 6. Polariton mode conversion at the uncovered step vs. the covered step

In this section, we compare the $l = 0 \rightarrow 1$ polariton mode conversions from the two 51nm-17nm steps in the top part of the hBN terrace in Figure 1d, featuring a covered step edge (left) and an uncovered step edge (right). The two steps share identical thinner-side thicknesses and the same thicker side, but the uncovered one exhibits a steeper edge (Supplementary Figure 7a). This steeper geometry more effectively scatters the incident polaritons, resulting in a higher $l = 0 \rightarrow 1$ mode conversion rate and stronger fringe oscillations (Supplementary Figure 7b).

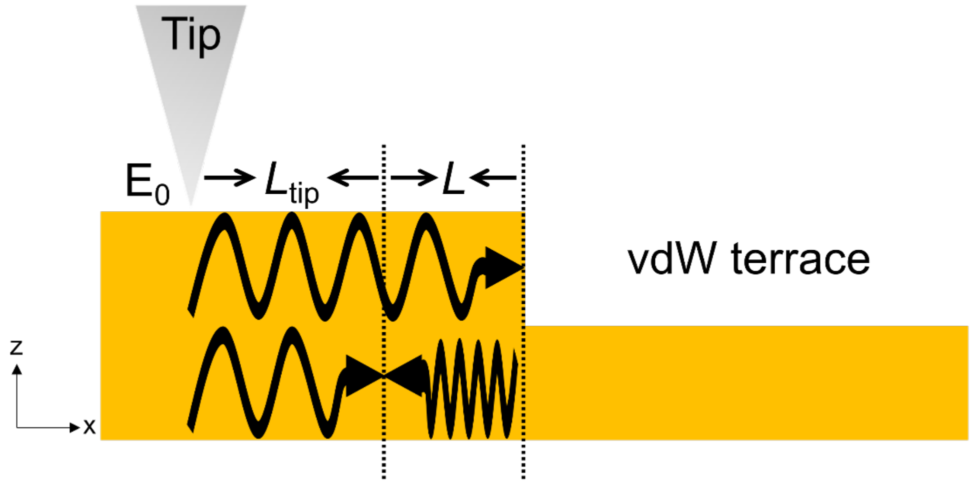

Supplementary Figure 1 | Standing wave interference mechanism in van der Waals (vdW) terrace. A schematic illustration for high-order polariton fringes formed by interference between the converted first-order polariton (bottom short-wavelength black arrow) from zeroth-order polaritons (top long-wavelength black arrow) and the newly launched zeroth-order polaritons (bottom long-wavelength black arrow).

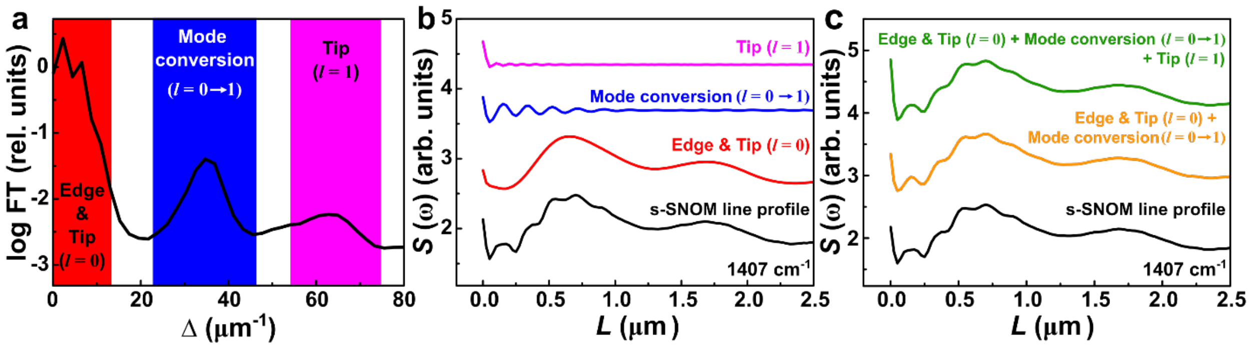

Supplementary Figure 2 | The decomposition of scattering-type scanning near-field optical microscopy (s-SNOM) line profile by Fourier Transform (FT) analysis. a, The FT spectrum of the s-SNOM line profile (reproduced from Figure 2c). Red, blue and pink rectangles mark the resonant features. **b,** The inverse FT transforms of the filtered regions in (**a**). The black curve shows the s-SNOM line profile (reproduced from the red curve in Figure 2a). **c,** Green: the sum of red, blue, and pink components in (**b**). Orange: the sum of red and blue components in (**b**). Black: the s-SNOM line profile. Frequency: 1407 cm^{-1}.

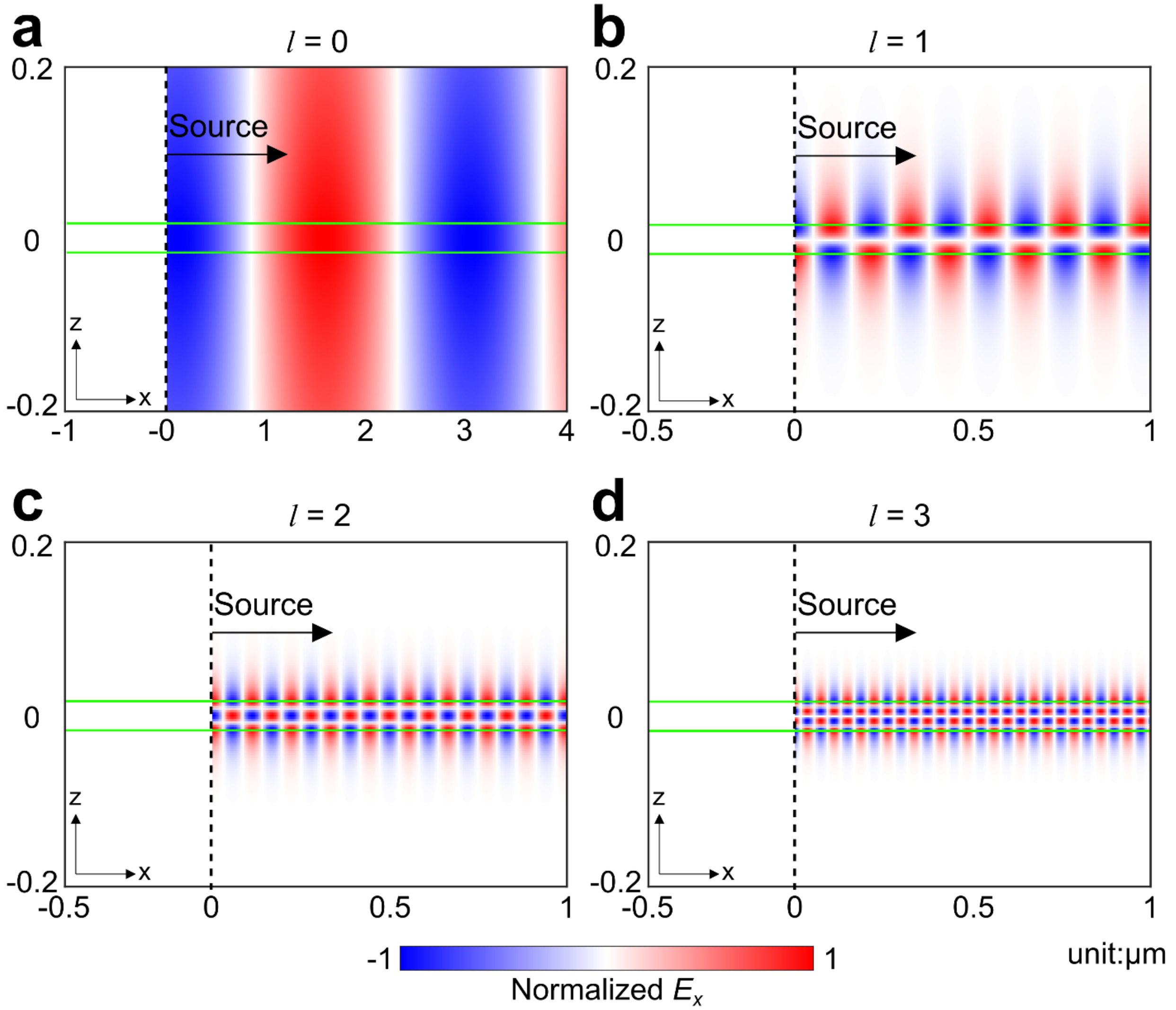

Supplementary Figure 3 | Numerically calculated electric field distribution of hBN. a-d, The normalized *x*-component of electric field (E_x) distribution propagating in positive *x*-direction at black dashed line for (**a**) zero-order, (**b**) first-order, (**c**) second-order, and (**d**) third-order mode is injected as the source to an infinitely long single slab hBN (green box).

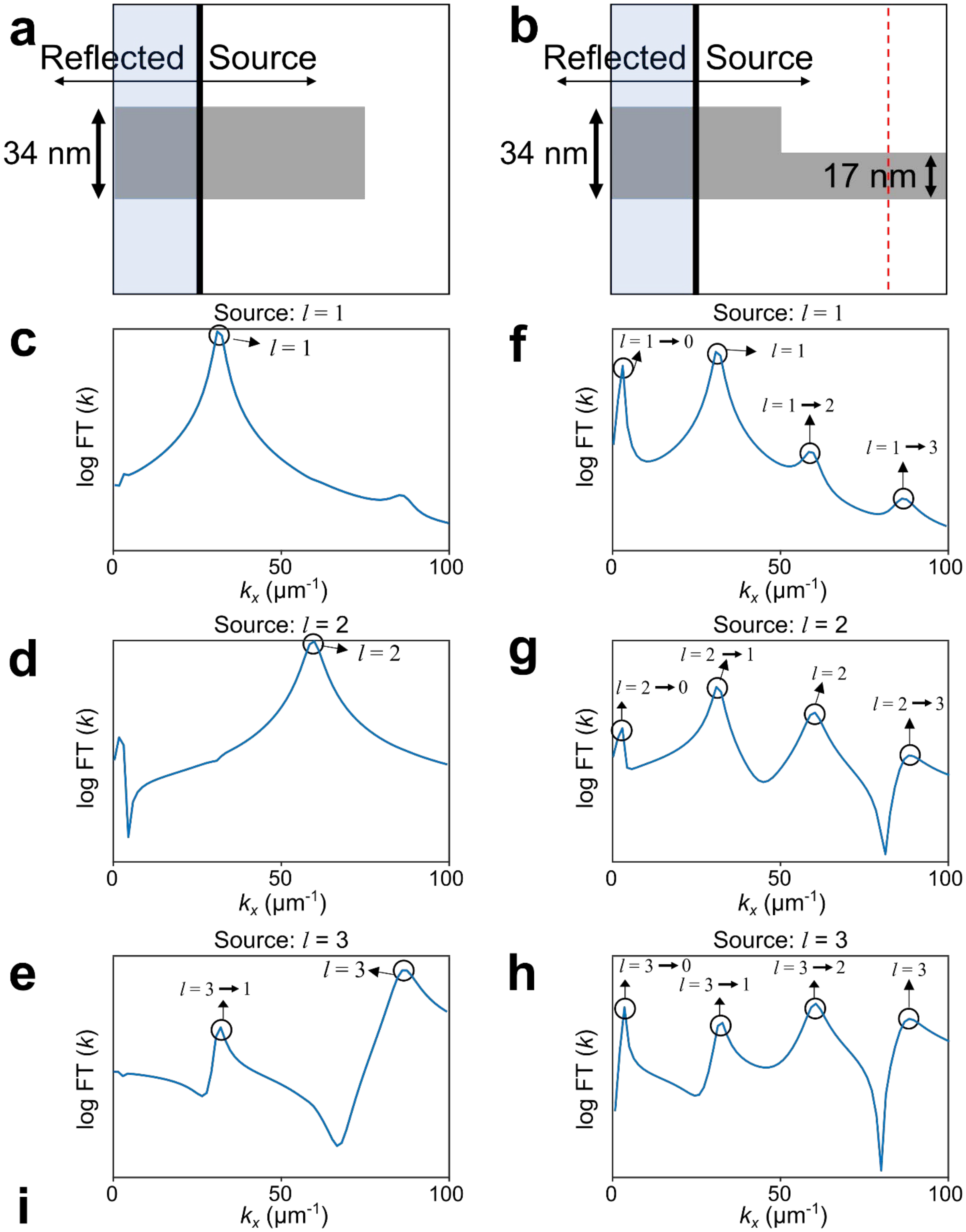

i

$T_{00} = 0.78$	$T_{10} = 0.13$	$T_{20} = 0.00$	$T_{30} = 0.00$
$T_{01} = 0.00$	$T_{11} = 0.14$	$T_{21} = 0.59$	$T_{31} = 0.22$
$T_{02} = 0.00$	$T_{12} = 0.01$	$T_{22} = 0.01$	$T_{32} = 0.18$
$T_{03} = 0.00$	$T_{13} = 0.00$	$T_{23} = 0.00$	$T_{33} = 0.02$

Supplementary Figure 4 | Finite-difference frequency-domain (FDFD) simulation results of polariton mode conversions with the input of high-order polaritons with l = 1, 2, and 3. **a**, **b,** Schematic illustration of the symmetric (**a**) simple slab and asymmetric (**b**) step-shape edge structures. The injected source launches hyperbolic polaritons at the solid black line and propagates along the $+x$ direction. The hyperbolic polaritons are reflected back at the edge and are finally analyzed at the left side of the source (light blue regions). **c-e, f-h,** FT spectra of the reflected field in the symmetric (**c-e**) and asymmetric edge (**f-h**) structures at 1407 cm^{-1} when (**c, f**) first-order mode (odd mode), (**d, g**) second-order mode (even mode), and (**e, h**) third-order mode (odd mode) are injected into the system. The order of the mode for each peak is pointed out in each figure. **i,** Transmissivity (T_{ij}) of different modes in a step-shaped edge (asymmetric structure). The transmitted fields are recorded on the right side of the hBN terrace (red dashed line) to calculate T_{ij}.

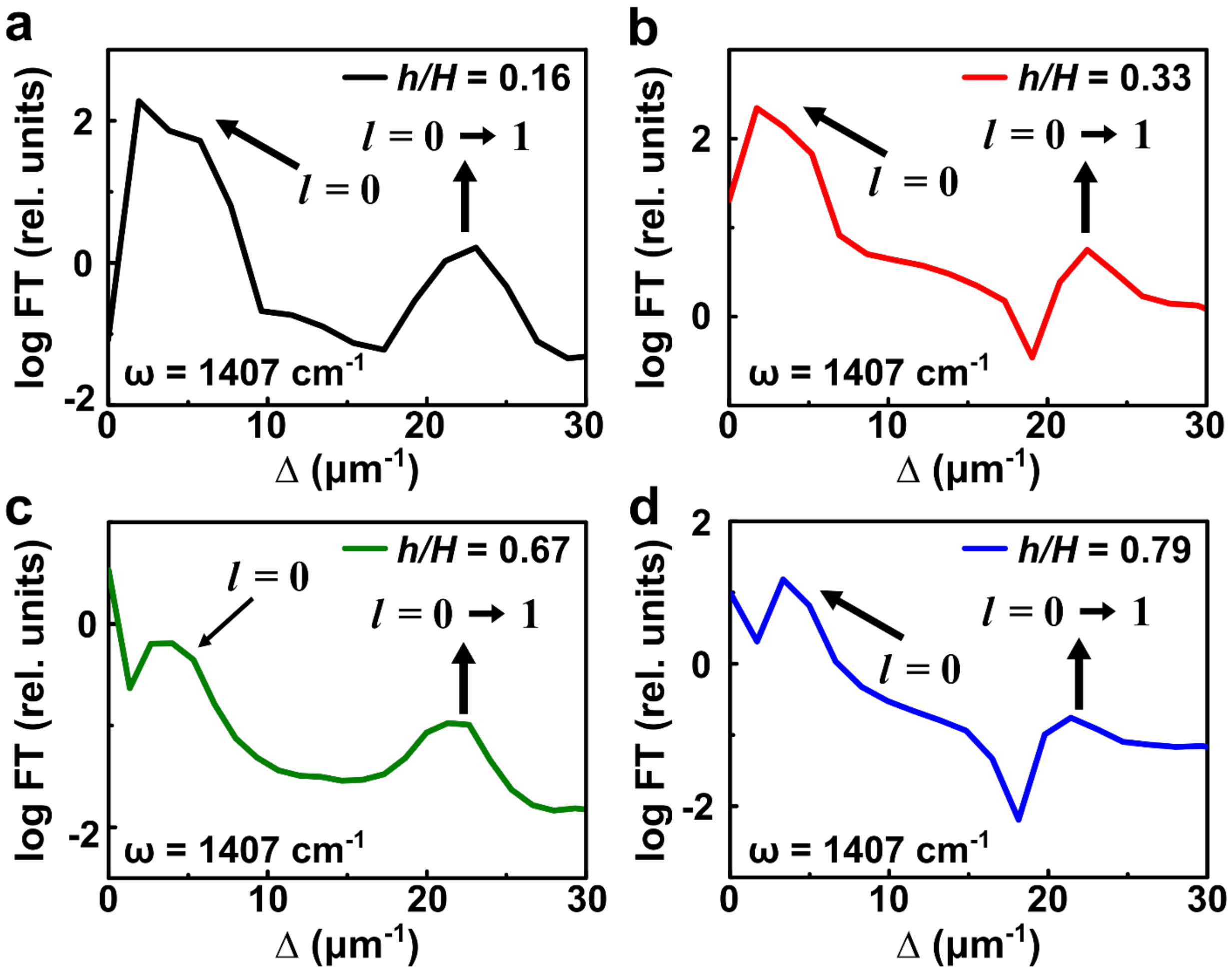

Supplementary Figure 5 | FT analysis of the step-size dependent s-SNOM data on polariton mode conversion. a-d, FT spectra of s-SNOM line profiles in Figure 5d with different step ratios *h/H* = 0.16 (**a**), 0.33 (**b**), 0.67 (**c**), and 0.79 (**d**).

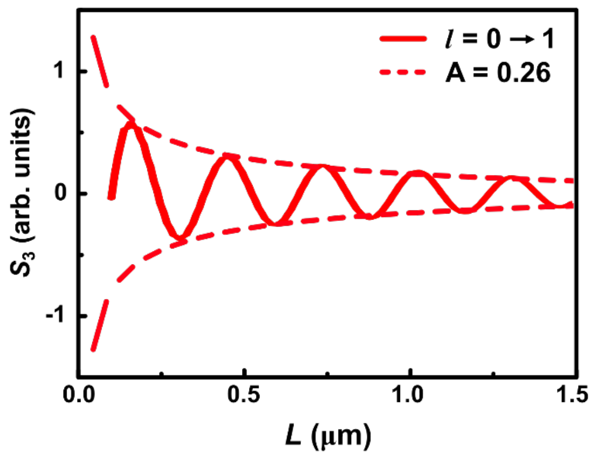

Supplementary Figure 6 | The extraction of the oscillation intensity of the *l* = 0 → 1 polariton. The oscillation intensity *A* is extracted by fitting the *l* = 0 → 1 s-SNOM line profiles with the envelope of a sinusoidal wave function Ae^{kL}, at a representative step ratio $h/H = 0.33$. IR frequency $\omega = 1407$ cm^{-1}.

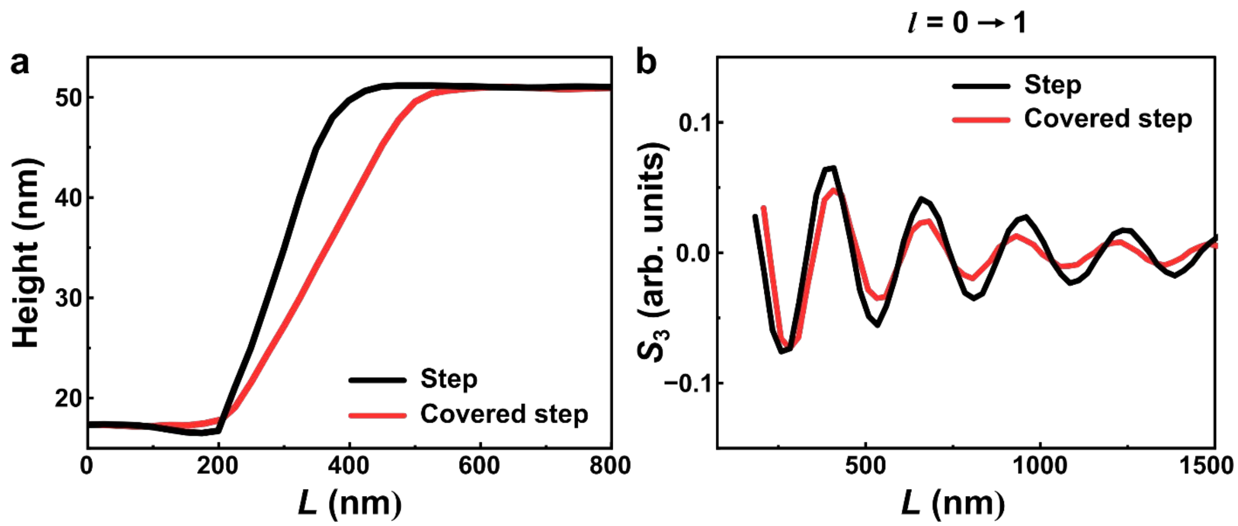

Supplementary Figure 7 | Polariton mode conversion at the uncovered step vs. the covered step. a, The AFM topography across the uncovered (black) and covered (red) steps from Figure 1d in the main text. **b,** the s-SNOM line profiles of the mode converted $l = 0 \rightarrow 1$ polaritons from the uncovered (black) and covered (red) steps. The two steps share the identical thinner side thickness, $h = 17$ nm, and the thicker side thickness, $H = 51$ nm. IR frequency $\omega = 1407\ \mathrm{cm}^{-1}$.

Supplementary References

1. Dai, S. et al. Tunable Phonon Polaritons in Atomically Thin van der Waals Crystals of Boron Nitride. *Science* **343**, 1125-1129 (2014).
2. Zhao, B. & Zhang, Z.M.M. Perfect mid-infrared absorption by hybrid phonon-plasmon polaritons in hBN/metal-grating anisotropic structures. *International Journal of Heat and Mass Transfer* **106**, 1025-1034 (2017).
3. Dai, S. et al. Efficiency of Launching Highly Confined Polaritons by Infrared Light Incident on a Hyperbolic Material. *Nano Letters* **17**, 5285-5290 (2017).
4. Dai, S. et al. Subdiffractional focusing and guiding of polaritonic rays in a natural hyperbolic material. *Nature Communications* **6**, 6963 (2015).
5. Woessner, A. et al. Highly confined low-loss plasmons in graphene-boron nitride heterostructures. *Nature Materials* **14**, 421-425 (2015).
6. Dai, S.Y. et al. Hyperbolic Phonon Polaritons in Suspended Hexagonal Boron Nitride. *Nano Letters* **19**, 1009-1014 (2019).
